# Training with High Fidelity Simulation in the Care of Patients with Coronavirus—A Learning Experience in Native Health Care Multi-Professional Teams

**DOI:** 10.3390/healthcare9101260

**Published:** 2021-09-24

**Authors:** Andrés Rojo-Rojo, Maria Belén Soto-Castellón, Juan Antonio García-Méndez, César Leal-Costa, Maria Gracia Adánez-Martínez, María José Pujalte-Jesús, José Luis Díaz-Agea

**Affiliations:** 1 Faculty of Nursing, Universidad Católica de Murcia (UCAM), Guadalupe, 30107 Murcia, Spain; arojo@ucam.edu (A.R.-R.); mbsoto@ucam.edu (M.B.S.-C.); jluis@ucam.edu (J.L.D.-A.); 2 Emergency Management of the Region of Murcia, Murcia Health Service, 30005 Murcia, Spain; jagmendez@ucam.edu; 3 Faculty of Nursing, Universidad de Murcia (UM), Campus de Espinardo, 30100 Murcia, Spain; 4 Faculty of Medicine, Universidad de Murcia (UM), Campus de Espinardo, 30100 Murcia, Spain; g.adanez@um.es

**Keywords:** simulation, coronavirus, high fidelity simulation training, COVID-19, interprofessional education

## Abstract

The training of emergency and intensive care teams in technical and non-technical skills is fundamental. The general aim of this study was to evaluate the training of various professional teams with simulations based on the care of COVID-19 patients using Zone 3 simulations (native emergency medical services and intensive care units-ICU teams) in the Region of Murcia (Spain). A mixed pilot study was designed (qualitative/quantitative) comprised of three phases: Phase 1: detection of needs (focus groups), Phase 2: design of simulation scenarios, and Phase 3: training with high-fidelity simulation and evaluation of competences. The results were used to determine the real training needs of these health professionals, which were used to design four simulation scenarios in line with these needs. The team competences were evaluated before and after the training session, with increases observed after the training sessions, especially in non-technical skills such as communication. Training with zone 3 simulation, with multi-professional native emergency and intensive care teams who provided care to patients with coronavirus was shown to be an effective method, especially for training in non-technical skills. We should consider the training needs of the professionals before the start of any training program to stay one-step ahead of crisis situations.

## 1. Introduction

During the current world pandemic situation provoked by the COVID-19 virus [1,2], one of the most controversial aspects was the safety of the health professionals who provided care to sick patients [3]. When discussing safety, we are not only referring to physical safety, but also to psychological safety, which also became compromised, with important repercussions in the mental health of health workers [4,5]. In this sense, the frontline health workers have had to face a great challenge which has had unprecedented consequences, given the duration and persistence of this pandemic [6,7].

Health professionals were at a high risk of becoming infected during the initial phase of the coronavirus SARS Cov2 pandemic, especially before its transmission dynamics had been completely deciphered. The health personnel infection rate was high worldwide at the beginning of the pandemic, and in Spain, this was very worrisome [8,9]. The infection figures were around 120,000 infections and 63 deaths due to COVID-19, with the greatest number found during the “First Wave”.

In second place, the fear of becoming infected became a feeling of being fed up, psychological exhaustion, and symptoms compatible with post-traumatic stress [10]. This had repercussions on behaviors such as an increase in the consumption of toxic compounds (tobacco and/or alcohol), anxiolytic drugs, loneliness, and the low capacity to communicate experiences and feelings [11].

All of these situations not only had an effect on the emotional structure, but on the cognitive functions of these health professionals as well, affecting their decision-making processes in the clinical environment, thereby implying a risk for the patients [12,13]. Minimizing the psychological impact of the current pandemic due to COVID-19 in health care workers is a special challenge for health care systems worldwide.

Among the main causes for these figures, we find the lack of personal protection equipment (PPE) at the national and international level, as well as the infection of patients with slight symptoms or asymptomatic patients who sought help at the health services centers. One of the variables, which had an influence on the initial confusion in the management of critical COVID-19 patients, was the scarce training of the health professionals associated to how to address the different situations that arose. We believe that aside from the indispensable barriers provided by PPE, previous and adequate training, with technical and non-technical skills in the care of these patients, would have had a favorable repercussion on the health of the health care personnel.

Aside from training on clinical skills, training centered on the improvement of non-technical aspects, such as communication skills, should also be planned [14]. There are numerous cognitive and psychological variables which have an influence on the manner in which health professionals deal with direct threats to their person during their caregiving tasks. Training can be provided on these aspects [15,16,17,18], especially in an experiential and reflective manner through clinical simulation [19]. Technical skills are those related with procedures or techniques. For example, the dosage and how to administer a sedative, knowing how to interpret an electrocardiogram, recognizing the symptoms of a specific disease, performing a maneuver such as intubation, etc. On the other hand, the non-technical skills are related with behavior depending on the clinical situation, including interpersonal relations and cognitive processes that could provide support to technical and clinical skills. Within the non-technical sphere, we include aspects related with communication, teamwork, leadership, management of emotions, awareness of the situation, and the making of decisions, for example.

At present, learning with simulation is one of the most important types of training in many healthcare professions, given its experiential and reflective character [20]. However, special emphasis should be given to its efficacy when safeguarding the safety of the professionals, and especially the patient [21,22].

Numerous simulation models exist; however, training according to zones [23] allows for the gradual training of skills and the increase in knowledge. In zones, 0, 1, and 2 training is done on the skills and competences of students and professionals, who train in environments with an increasing level of noise. However, starting with zone 3, training can be conducted with multidisciplinary teams of native health professionals (belonging to real-world environments) in simulated environments that are remarkably similar to those in which they conduct their daily activities, and with cases associated to their specialty, as is the case in the present study.

On the other hand, it should be highlighted that clinical simulation has provided good results in the increase of competences of students [24], as well as professionals or patients [25], and informal caregivers [26]. The training of health professionals based on the clinical simulation methodology has been used for a long time, with successful results observed [27,28,29,30].

In all of these experiences, the increase in the simulation participant’s competence have been reported, which results in better care provided a posteriori to the patients. Both the use of simulated environments (zone 3) and the design of “simulation in situ” experiences improve the performance standards of the participants [30].

In addition, at present, experiences with clinical simulation have become a strategy for the training of professionals who have to care for COVID patients, given the success they have shown [31,32,33,34].

In this sense, organizations such as the European Society of Paediatric and Neonatal Intensive Care urges the use of training strategies based on clinical simulation, to efficiently increase the professionals’ competences associated to COVID [35]. In the context of the pandemic, some health institutions have wagered on training based on simulation with their clinical staff, both veteran and new members [36].

The general aim of this study was to evaluate the training of various professional teams with simulations based on the care of COVID patients using zone 3 simulations (native Emergency Medical Services (EMS) and hospital Intensive Care Unit (ICU) teams in the Region of Murcia (Spain). The specific objectives were: To determine the training needs of multi-professional health care teams with respect to technical and non-technical skills associated to the care of COVID-19 patients; to design simulation scenarios adjusted to the needs of the participants; to put into practice high-fidelity clinical simulation scenarios with native workers (zone 3); and to analyze the acquisition of technical and non-technical competences during the simulated experiences.

## 2. Materials and Methods

### 2.1. Design

**Figure 1:**
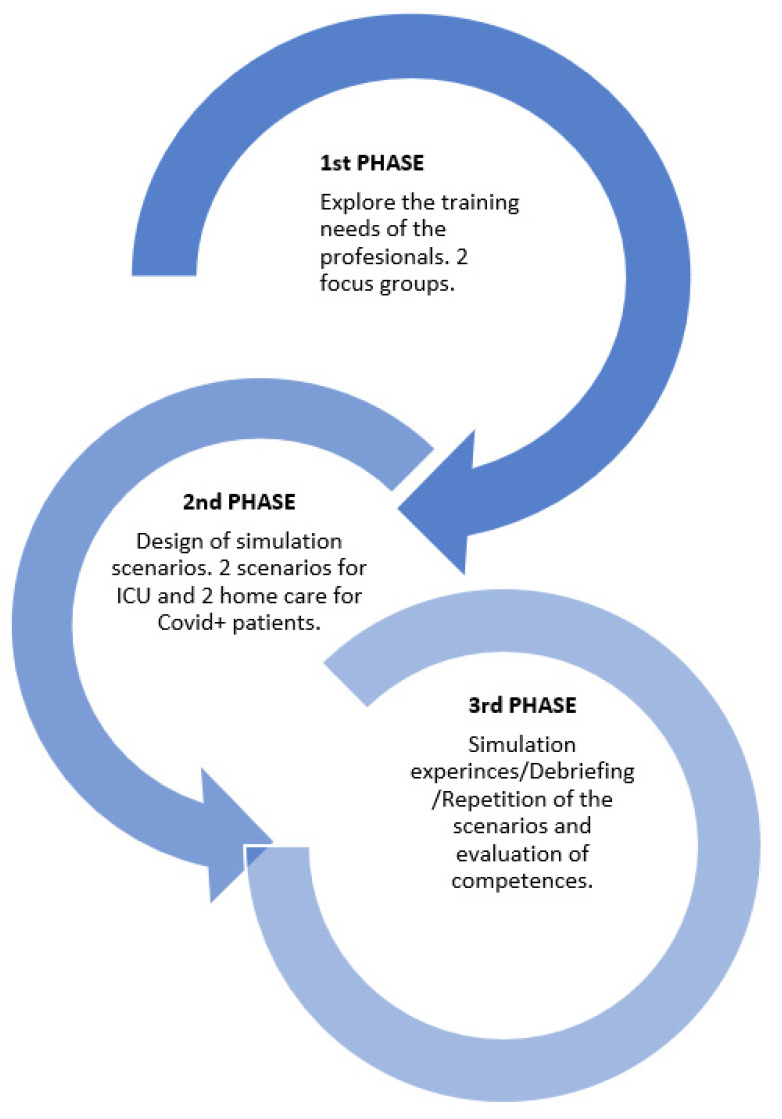
Study phases.

A mixed pilot study (qualitative/quantitative) was designed, comprised of three phases (Figure 1). In the first phase, two focus groups of professionals were convened to discuss aspects related with the preparation of professionals for facing the health crisis due to the pandemic and the specific training needs. The results from the analysis of the focus groups helped the research team to decide on the competences to work on and to design a series of clinical simulation scenarios based on the different environments in which the COVID-positive patients would be treated. In addition, an evaluation tool was created for each scenario, which was adapted to the competences that were to be acquired with the training. This was the second phase of the study. In the third phase, a pre-post intragroup (without a control group) quasi-experimental study was conducted, in which health care professional teams were trained in the clinical simulation installations at the Catholic University of Murcia (Spain). The competences acquired by the health professionals were evaluated before and after the training with simulation. The contents of the debriefing sessions from each scenario were also analyzed qualitatively.

### 2.2. Participants

Two emergency medical services (EMS) teams were selected, as well as two public health intensive care unit (ICU) professional teams from the Region of Murcia (Spain). Non-random, snowball intentional sampling was utilized. The volunteers had to be willing to take part in the simulation experiences without prior knowledge of the scenarios, they had to be real teams with a long-term history (more than 2 years of experience), and must have exerted their caregiving labor during the pandemic. For the simulation experiences, the participating teams selected are shown in Table 1. For the focus groups, the selection was broadened to 12 participants in the case of the intensive care unit group, whose characteristics are detailed in the procedure section.

### 2.3. Procedure

The study was conducted between the months of September and December 2020, at the UCAM installations (area of simulation). The procedure for each phase is explained below:

Phase 1: This phase took place on the first week of September 2020. Two different focus groups were organized: a group of six EMS professionals and another group of 12 ICU professionals. The focus groups were moderated by an expert in qualitative methodology and were designed having in mind that the focus of discussion would be centered on the following subjects: Pandemic/Subjective impression/Self-evaluation of actions during the pandemic/Aspects to be improved in caregiving/Training needs. The two focus groups sessions took place at different times (Table 2). Both groups had the same structure: opening of the session with a welcome and presentation of the research project; presentation of the participants themselves; clarifications about the dynamics of the discussion; establishment of a spoken contract with the group, highlighting the ethical aspects of the research and the interaction processes; guidance of the debate centered on the subjects to be discussed; synthesis of the previous phases; closure of the session, and acknowledgement of the participants. Due to the risk of infection, all the safety measures were used (distancing, use of mask, ventilation of the room, and use of disinfecting gel before and after the meeting).

Phase 2: It was carried out between the second week of September 2020 and the first week of October 2020. With the information obtained in the focus groups, four simulation scenarios were created (two for each group/one per team). The recommendations from the associated literature were considered for the design of the scenarios [37,38,39]. To evaluate each scenario, a tool was designed to evaluate the dimensions (due to their length). Each checklist organized the skills with a Likert-type scale with five response options: 1 = “never performed” and 5 = “always performed”.

For the EMS group, two scenarios at the patient’s home were designed.

In the first scenario, the team is coming back to base from a call at the city center and are inside the ambulance. At that moment, they receive a call from the Emergency Coordination Center with a new emergency from a nearby town (20 min away). The data provided are the following: 45-year-old male with chest pain, who was infected with COVID with slight symptoms. The learning objectives for this case were: Heart attack code in a COVID context; Intubation in COVID context and Problem resolution and decision making in a complex context. The non-technical skills that were worked on were: Transfer of complex information (SBAR method); Problem resolution technique from D Zurilla and Goldfried [40].

In the second scenario, the team is having lunch. It is 1:35 pm, and they have just sat down to eat when they get the call: Woman, 67 years old, with dyspnea and fever. She lives alone and talks daily with her daughter Alicia, who is on her 36th week of pregnancy. Today, during a video call with her daughter (1 pm), the daughter saw her mother worse and went gone to see her, calling 112 afterwards. The 112 ambulance driver (who will be an actor) will hinder the work of the participants and will have a disruptive behavior throughout the scenario. The learning objectives in this case were: Assertiveness techniques; Level of severity in respiratory failure; Indication of transfer to hospital. The non-technical skills were: Assertiveness and assertiveness techniques, differentiating depersonalization behavior due to burn-out (driver) from a reaction due to fear (daughter), and personal self-control.

For the ICU group, two intensive care units scenarios were designed. In the first case, a scenario was planned about the administration of a common IV medicine to a patient who was in the prone position with full-blown hypoxia and critically sick. When the nurse is present, the patient suffers severe hemodynamic deterioration with sustained hypotension and cardiorespiratory arrest.

This scenario counted with the participation from the nurse who was with the patient and the ICU doctor, along with nursing assistant support. The learning objectives for this case were to perform advance life support on a patient in the prone position (according to the ERC guide), prioritizing safety, and effective communication within the team.

In the second scenario, the multidisciplinary team, composed by an ICU doctor, nurse and nursing assistant, had to be responsible for an admission of a COVID+ patient in a situation of full-blown hypoxia, conscious, agitated, with spontaneous breathing, with a pattern of altered breathing. The health care team at the emergency entrance door (doctor and nurse) transfers the patient.

The learning objectives for this case were: the transfer of a COVID+ patient between the Emergency department and ICU, the initial management of a COVID+ patient, management of an agitated patient who rejects treatment, and the use of non-invasive mechanical ventilation (NIMV) on a COVID+ patient.

Along with the design of the scenarios, an ad hoc evaluation tool was created by the research team, which served to evaluate the behavior of the participants during the simulation session. To provide content validity, the dimensions and selected items were submitted to the judgment and discussion of a panel of experts. For each type of scenario, a different panel of experts was used; one for EMS scenarios; another for ICU scenarios. Each panel was comprised of three experts, at the teaching, care and scientific levels. The inclusion criteria as an expert were the following: to be an active professional in the study area for a period of no less than 5 years; have training experience in the area studied; be a university professor/clinical simulation instructor. Regarding the evaluated aspects, in each selected dimension, the following characteristics were evaluated with respect to the items issued: sufficiency (is the number of items sufficient/insufficient? Is there redundancy? Would any item be added?); clarity (is the item hard to understand? Do you require clarification?); coherence (does the item have a logical relationship with the action?); and relevance (can the item be eliminated without affecting the measurement of the dimension?). As conclusions of the two panels of experts, the registration scales were issued for each of the four designed scenarios.

The evaluation tools had good content validities (CVI = 1), were created through consensus after various meetings, and included dimensions with a variable number of items according to scenario, to be posteriorly evaluated by independent observers.

Phase 3: Simulation sessions were planned for each group. The groups A (EMS) and B (ICU) were both subjected to two different simulation scenarios, which were repeated twice. It was carried out on different dates for each of the groups studied. EMS scenarios were performed on the first week of November 2020; the scenarios with the ICU team were carried out on the last week of November 2020. For the group A simulation, a teaching ambulance was utilized, which was equipped exactly as the one used by Emergency services in the Region of Murcia, and a simulation room, which re-created the patient’s home. For the development of both scenarios, a high-fidelity simulation manikin (CAE ARES Emergency Care Manikin^®^) and a trained actress, who played the role of a family member, were utilized. In the case of group B (ICU), the scenarios took place in the simulation room, which re-created an environment of intensive care at a hospital with a high-fidelity simulator (Laerdal^®^ SimMan Essential).

In each group studied, six participants were selected according to intentional criteria of volunteerism and opportunity and minimum experience of 5 years in the department of 5 years, having recently experienced situations with COVID patients in the real world. In the composition of the teams, the work structure of the units themselves was maintained. Thus, the EMS team was composed of six members (two doctors, two nurses and two health emergency technicians). The intensive care team was composed of 6 other members (two doctors and four nurses).

On the day of the simulations, the participants became familiarized with the material, the room, the ambulance, and the surroundings. Afterwards, a psychologically safe environment was created so that the participants felt comfortable, by resolving the doubts that could arise after the explanation of what they were about to experience. Ice-breaking activities took place before the start of the simulations.

After this, we established two work teams (Team 1 and Team 2 for group A-EMS; Team 3 and Team 4 for group B-ICU) to perform in a simulated scenario. This team remained stable in the Pre and Post situation.

Afterwards, the initial guidelines were provided, and each team received brief instructions about the case (Briefing) in SBAR (Situation, Background, Assessment, and Recommendation) format. Next, the simulation scenario took place. The actions of the teams were recorded using two video cameras (one in front of the patient, and another set-up at an angle). After the simulation, a structured debriefing session took place (using the Gather/Analyze/Summarize format) [41]. After the debriefing session, each team repeated the same scenario a second time; in this manner, we obtained the Pre and Post debriefing action. The characteristics and duration of the scenarios are shown in Table 3.

### 2.4. Data Analysis

The data from the focus groups and discourses generated in the case debriefing sessions were collected as videos and transcribed verbatim. Afterwards, they were subjected to content analysis and categorization/codification to obtain qualitative data. Two independent researchers who utilized consensus to define the subjects, categories, and sub-categories that served as the basis for the design of the scenarios (Phases 1 and 2) performed the analysis. The conversations generated in each debriefing session served for contextualizing the learning and the quantitative data. The content of the debriefings in each case was transcribed, codified, and analyzed.

As for the quantitative results associated to the acquisition of competences after the simulation experience, an observational method of analysis was used, in which 10 independent expert observers (five different experts to evaluate each group, and who were not part of the research team) evaluated each scenario blindly (without knowing if the video they were evaluating corresponded to the initial simulated case or the repetition after the debriefing session). The experts were chosen for having 5 years of experience in an intensive care unit (ICU), and emergency medical system (EMS), respectively).

To ensure an objective evaluation, random numbers were assigned to the videos. The experts were chosen for having experience in the teaching of patient care through clinical simulation at the university, and for having broad experience in the area of care from the scenarios, (the experts who evaluated the scenarios from group A were not the same as for group B, as the areas of knowledge were different). These experts evaluated the acquisition of skills by the teams. To ensure that the evaluations were consistent, the experts were instructed on how to use the evaluation tool.

The expert’s evaluations were entered into a Microsoft Excel^®^ sheet, which was used to build a database with the SPSS © v21 program (Statistical Package for the Social Sciences, IBM Corp. Release 2012) to process the data.

The main outcome of the research was the post-test scores obtained for the dimensions assessed such as protection, coping, and communication, among others. To analyze the data, different statistical tests were utilized: descriptive statistics (mean and standard deviation) for the quantitative variables, and frequencies and percentages for the categorical ones.

As most of the measurements of the results did not have a normal distribution, the data before and after the interventions were subjected to a bootstrap analysis [42] and were presented as mean and CI 95%. The differences between the variables were analyzed with Student’s t-test with a Bonferroni correction for multiple comparisons. The data were considered statistically significant at *p* < 0.05.

To assess the internal consistency of the items amongst themselves, Cronbach’s Alpha coefficient was calculated; a minimum value above 0.7 was considered as an acceptable Cronbach’s Alpha value.

In addition, the effect size was calculated with Cohen’s D to evaluate the magnitude of the effect of the intervention, utilizing the values proposed by Ferguson [43], where 0.41 indicates a small effect, 1.15 a medium effect, and 2.70 a large effect. To estimate the reliability of the evaluation forms, the inter-observer agreement test was applied by calculating the intraclass correlation coefficient (ICC) [44,45]; its value was interpreted as a percentage of the variability of the score which was solely dependent on the variability between the subjects evaluated.

### 2.5. Ethical Considerations

During the study, all the applicable institutional and governmental regulations on the ethical use of human subjects were followed. This research complied with the requisites from the Declaration of Helsinki and was approved by the Ethics Committee from the Catholic University of Murcia (code: CE072002). All the participants were informed about their participation and signed a form indicating their informed consent and authorizing the recording of their actions in the simulation to video, and the dissemination of these recording for scientific aims.

## 3. Results

For its better understanding, the results of this study are presented organized by phases.

### 3.1. Phase 1

#### 3.1.1. Emergency Medical Services Team Focus Groups

After the analysis of the focus group, four main categories and 12 sub-categories were obtained (Table 4) associated to the dimension training/entertainment. The data related to other dimensions (such as the criticism about the management of the pandemic, or organization matters and negative experiences at the local level), which were also discussed, are not presented in the present work, as they were not part of the research objective.

#### 3.1.2. ICU Teams Focus Groups

In this group, three categories and six sub-categories associated with training were obtained, as shown in Table 5. These categories were remarkably similar to those obtained in the EMS group. The data showed the need to train on technical (related with teams and procedures) as well as non-technical (related with the management of emotions or communication, especially bad news) skills.

### 3.2. Phase 2

As for aspects to be discussed in the debriefing session for the first EMS scenario, these were contraindications of the thrombolytic drugs and CPR maneuvers in a COVID context. The dimensions were: Protection (personal and environmental), with six items that could be evaluated; Clinical management of the scenario, with nine items that could be evaluated, and Effective communication with seven items.

To evaluate the second EMS scenario, each checklist organized the learning skills according to the different interventions, and evaluated the skills with a Likert-type scale. The dimensions were: protection (personal and environmental)/comfort with nine items to be evaluated; clinical management of the scenario, with five items to be evaluated; and management/control of a disruptive profile, with four items that could be evaluated.

*To evaluate the ICU first scenario, each checklist organized the learning skills according to the different interventions, and evaluated the skills with a Likert-type scale with 5 response options: 1 = “never performed”, and 5 “always performed”. The dimensions were: perform advanced life support on a COVID+ patient in the prone position; protection; (personal and environmental) effective communication, with a total of 24 items to be evaluated.*

### 3.3. Phase 3

The results of the dimensions showed an increase in the mean score obtained when the simulation scenario was repeated. The results of the dimensions for each scenario (initial and repeated) are shown in Table 6.

For the ICU scenarios, the dimensions which were statistically significantly different were “protection” (Figure 1) in the scenario with the agitated patient and “communication” in the ALS in prone position scenario. Within the “protection” dimension, significant differences in the initial and final evaluations were observed in items 5 (*t* = 2.99, *p* = 0.04, CI 95% 0.16–4.24) and 7 (*t* = 3.14, *p* = 0.04, CI 95% 0.18–3.02). In the dimension “communication”, the following items showed significant differences between the initial and final scores: item 4 (*t* = 3.16, *p* = 0.034, CI 95% 0.12–1.88) and 6 (*t* = 3.09, *p* = 0.037, CI 95% 0.18–3.42).

In the EMS scenarios, the dimensions in which differences were observed were those for the CPR scenario, the dimension “communication”, with a small effect size. Within this dimension, items 3 “Clear communication is established in the team” (*t* = 3.87, *p* = 0.037, CI 95% 0.18–3.42) and 6 “Order and control of the situation is maintained” (*t* = 3.207, *p* = 0.033, CI 95% 0.16–2.23).

On the other hand, in the scenario with the patient who had breathing difficulties, significant differences were found in all the dimensions: “protection”, with a medium effect size (in the items 2 “Good practices in protection (Do not touch the face, or the mask, place the material in a clean area...)” [*t* = 3.21, *p* = 0.03, CI 95% 0.16–2.24], item 4 “Ensure that the patient wears a surgical mask [*t* = 2.99, *p* = 0.04, CI 95% 0.16–4.24 ], and item 8 “Offer comfort/comfort to the patient” [*t* = 6.33, *p* = 0.000, CI 95% 1.12–2.88]), “disruption control” with a medium effect size (item 1 [*t* = 3.65, *p* = 0.02, CI 95% 0.48–3.52]), item 2 “Limit contact between family member and disruptive profile” [*t* = 3.54, *p* = 0.02, CI 95% 0.52–4.28]), item 3 “Avoid expressions of impatience-anger” [*t* = 2.99, *p* = 0.04, CI 95% 0.16–4.24]), item 4 “Maintain a calm attitude” [*t* = 4.33, *p* = 0.01, CI 95% 0.93–4.27]), “communication” with a medium effect size (item 2 “Professionals speak with a calm attitude” [*t* = 3.21, *p* = 0.03, CI 95% 0.16–2.24], item 3 “Verbal restraint is performed with family member: Do not be provocative, be peaceful, reassuring, and friendly” [*t* = 2.45, *p* = 0.07, CI 95% 0.16–2.56], item 6 “Establish certain limits to generate a favorable environment for communication and thus avoid interruptions, noise, and chaos” [*t* = 4.90, *p* = 0.02, CI 95% 0.70–3.30], item 7 “Establish proper verbal contact: Be concise, speak plainly and simply” [*t* = 4.81, *p* = 0.01, CI 95% 0.76–2.84], item 8 “Ensure that only one person interacts with the patient” [*t* = 2.45, *p* = 0.07, CI 95% 0.08–1.28]) and “clinical management” with a medium effect size (item 4 “Application of immediate respiratory support (oxygen)” [*t* = 3.77, *p* = 0.02, CI 95% 0.58–3.82] and item 5 “Comprehensive and structured approach to care” [*t* = 5.88, *p* = 0.00, CI 95% 1.16–3.24).

The Cronbach’s Alpha values for the EMS scenarios were α = 0.890 for the CPR scenario, and α = 0.923 for the agitated patient. As for the ICU scenarios, these following values were obtained: α = 0.953 and α = 0.95 for the CPR and agitated patient, respectively.

The ICC obtained for the ICU scenarios were: agitated patient, ICC = 0.904 (very good), ALS scenario = 0.61 (substantial). The values for the EMS scenarios were: for the ALS case, ICC = 0.788 (substantial), and for the patient with respiratory difficulty case, ICC = 0.72.

**Figure 2:**
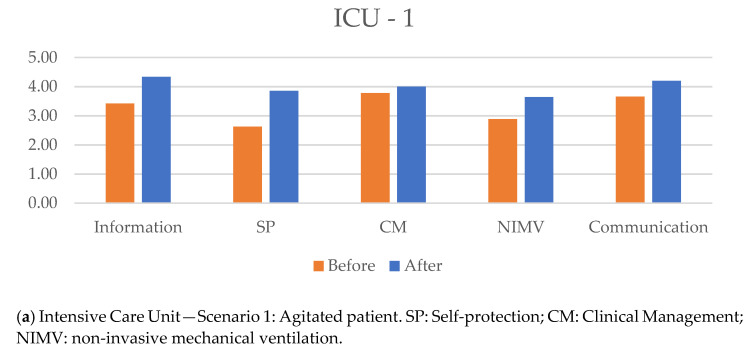

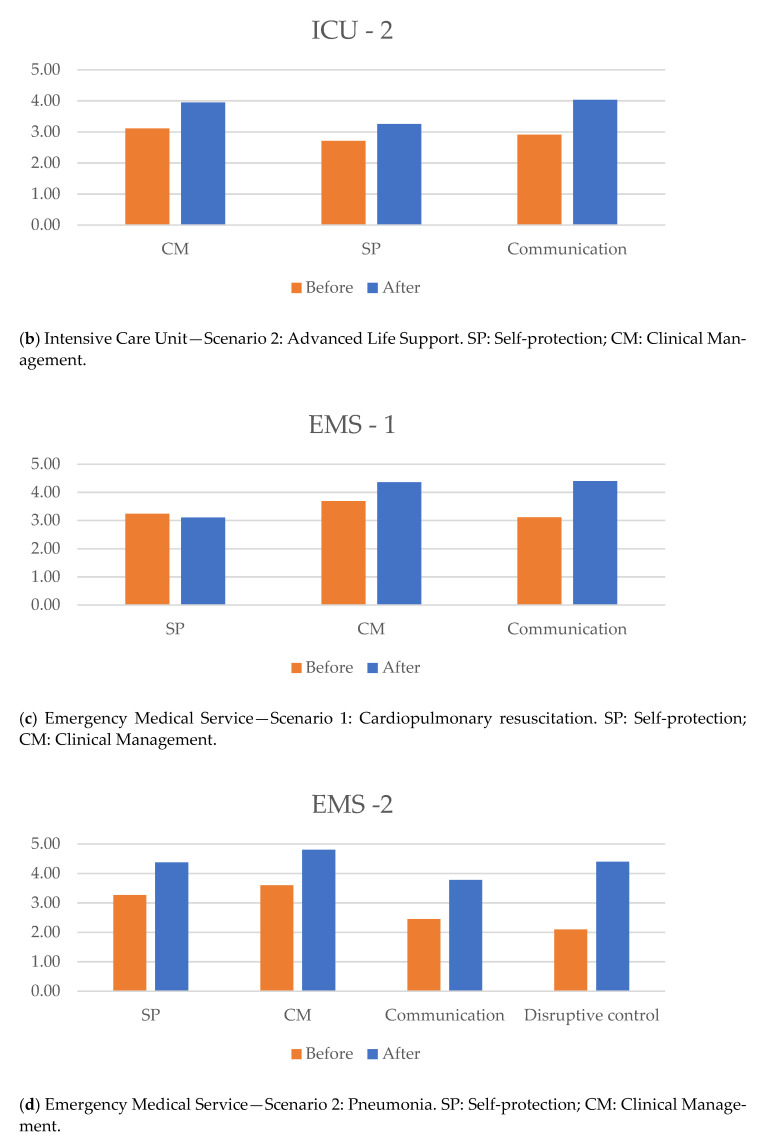
Overall score for each dimension for the different scenarios in the initial and repeat evaluations.

Figure 2a–d show the differences in the overall scores of the dimensions for each scenario in the initial and repeated evaluations.

## 4. Discussion

Considering the results found, the following discussion elements are now presented. The primary concern is the need to count with health professionals when creating a training plan according to their needs. Usually, the training plans in health centers and official training centers do not consider the real needs of the participants. As for the pandemic due to COVID-19, the training plans were at first scarce and improvised. The health professionals were almost self-learners in the acquisition of knowledge and the skills needed to face this challenge.

Facing a novel health crisis, such as a pandemic, is a challenge for scientists and organizations responsible for the health of the population. These viral outbreaks have historically provoked the need for the global communities to rapidly plan, prepare, and ensure health care. Nevertheless, this immediacy and novelty of the pandemic–epidemic did not give us enough time or foresight to consider the training needs. In the present pandemic (especially at the start), information and training were provided to health professionals in an improvised manner, with much of the information lacking in certainty.

In this sense, the practice of technical skills, as well as the application of procedures (maneuvers in the management of the airway, ventilation therapies, etc.), and the use of personal protection equipment were urgent matters, in detriment to the non-technical competences, such as communication skills or caring for colleagues [46].

The immediacy and the improvisation of the specific training programs in technical aspects, experienced during the period of COVID in different health centers, goes against the recommendations according to evidence [47], which indicate that the “foresight” through the programmed training of the personnel and teams contribute with minimizing the impact of these events on the quality of care. In this sense, training plans should be created that are adequate to “future” needs, based on prior events, or in deficiencies observed in the present, which can be projected into the future.

The needs detected in our study, at the technical and non-technical levels, could contribute with minimizing the negative impacts of these new epidemic “outbreaks” on health personnel. The possible improvements in the present and the future, in the training and the plans to improve the non-technical skills, are especially striking, with special emphasis on emotions themselves, together with the care and support of one’s colleagues.

The use of simulation as a multidisciplinary training strategy in contexts of emergencies has been successfully described [48,49,50], and our study corroborates the need for its implementation and its general use to work on all types of competences in a small amount of time.

In this study, we have learned that an adequate training plan must be based on an appropriate plan, which considers the needs of the professionals who will receive this training. A good strategy for collecting this information is the use of qualitative research techniques based on the production of opinions through the explicit use of interaction. We believe that the points of view on this phenomenon (pandemic and training needs), aside from being individual, are highly enriched if we consider the points of view produced during the interaction of different individuals in a focus group, aside from those that are produced in isolation.

In our study, the training needs were focused on both technical and non-technical skills. Therefore, the scenarios were designed to include contents that referred to the types of skills mentioned by the participants in the prior focus groups.

The use of clinical simulation allowed for experiential and reflective learning that was immersive in nature, within the context of caring for coronavirus patients. This element allowed the professionals to re-create possible scenarios from their real-world practice and to evaluate their actions and behaviors. It also allowed them to discuss their weaknesses and aspects to be improved in a safe environment. It is important to highlight that the safety offered by simulation training is beyond doubt. In a simulated environment, complex situations can be re-created which the participants can address by using their professional competences with complete safety (simulations are safe tools, for both the patients and the participants). This aspect is key when interpreting the results.

In agreement with the results from this study, the non-technical skills were the most impacted by the training with clinical simulation, as skills such as communication obtained statistically significant differences in most of the scenarios (EMS CPR *p* = 0.04; EMS breathing difficulty *p* = 0.015; ICU-ALS *p* = 0.05). In the results we can see how most of the dimensions showed higher scores after simulation training. In addition, the non-technical dimensions showed significant differences after training, with moderate effect sizes. Among the dimensions with the largest effect sizes after simulation were “Communication” and “Management Control of disruption”. We believe that the impact on the technical skills was lower, as the professionals had clinical experience in their respective areas, and perhaps the most novel learning results were associated with aspects such as communication or controlling a disruptive situation. For example, with respect to specific technical skills, statistically significant differences were not found in the scores of EMS-CPR protection (*p* = 0.67), and the posterior score was even lower than the previous score (although this was not statistically significant and the effect size was small and very close to 0.41, which is the minimum effect size that represents a “practically” significant effect.). This could be interpreted by considering that the technical competence of the health professionals was already high, and small changes during the repeated scenario could have affected the score of the evaluation.

The development of non-technical skills in the context of COVID was one of the strategies recommended for minimizing the impact of the experiences of the health personnel with this disease. Thus, this could contribute with the improvement of inter-professional relations, the recognition of roles, the situational awareness, the care of the members of a healthcare team, and the management of situations that are potentially harmful for the interests of the patient [51].

The optimization of the team’s actions through improved non-technical skills can lead to better decision-making, a greater efficiency, and greater adherence to safety standards, a greater resilience, and better results. The non-technical skills are cognitive and social skills that characterize high-performance individuals and teams. In this sense, the strategy utilized is useful [52] and pertinent, as it respects the realism and the psychological and social fidelity of the scenes.

It should be underlined that in our study, the training in non-technical skills was demanded, such as the transfer of patients between health care teams, communication, teamwork, and the management of a crisis, aspects which were not defined as important at the start of the pandemic by those responsible of training in the area of health.

In the literature [53,54], specific theoretical–practical frameworks exist for the management of non-technical skills by health care teams. The more popular models which stress the importance of teamwork and non-technical skills in emergencies and critical care are the Crisis Resource Management (CRM) [55,56] or TeamSTEPPS [57,58]. These skills are broadly defined as situational awareness, making of decisions, team communication, and leadership. In this sense, the strategies described by TeamSTEPPS have shown their usefulness in the management of these types of situations in intensive care contexts.

As for the clinical application/transfer of the results of the present research to a real health care environment, many aspects need to be discussed. In first place, we find the ethical dimension: a simulated learning environment is safer for the participants (especially during a pandemic). On the other hand, it is also better for the patients for the health professionals to train with manikins or actors, on skills or protocols so that they are safely utilized or applied in the future. We can also attest that more competent professionals can be obtained if they are trained on situations that they do not sufficiently control in the real world. The importance of non-technical skills has become evident in the present work, given that training on these skills is usually not provided or is shadowed by the technical skills. Thus, our results support the need to create multidimensional learning programs that take into account technical aspects (clinical skills and procedures), as well as non-technical skills (teamwork, assertiveness, leadership, etc.).

A reflection about the future perspectives of this research was provided by the recognition given by the health professionals to the suitability of this type of training, especially their desire to keep to training on non-technical competences within the functional work team. This proposal was kept in mind for a future line of work that will be directed towards the identification of a baseline of non-technical competences within a functional work team and the planning and implementation of training based on clinical simulation that covers the specific objectives defined for each group.

## 5. Conclusions

In general, the skills that were worked on with simulation with native emergency multi-disciplinary and ICU teams were improved, especially all the non-technical aspects such as communication. The demands from the care teams included technical and non-technical competences associated with the lack of training for dealing with the care of coronavirus patients. The scenarios designed according to the real training needs of the professionals allowed them to successfully work on these competences. Efficient training in a safe environment can be accomplished with the use of contextual high-fidelity simulation, through the insertion of personal elements and materials that grant realism to the re-created scene.

## 6. Limitations

Among the limitations, we find the low number of participants in the training planned, given the safety conditions defined by the risk of infection in meetings in which the students come in close contact with each other; more specifically, the need for social distancing during the study period. This made necessary the reduction in the number of participants in each space destined for the simulation. This also led to the establishment of measures such as a group composed of no more than six individuals, the use of adequate personal protection equipment in all the sessions (such as N95 masks), routine hand hygiene with the use of hydroalcoholic gel, and routine ventilation of the space every 15 min.

Another aspect to consider is about the measuring instrument. The use of an evaluation rubric format could be used as a way to avoid measurement distortion and decrease possible inter-observer variability. However, the internal consistency is adequate with the Likert-type format of the observation template.

## Figures and Tables

**Table 1:** Characteristics of the simulation study participants.

Participant Group	Professional Teams ^1^	Number of Participants and Professional’s Category	Workplace	Gender/Age
Group A (Emergency Medical Services)	TEAM 1	P1: Doctor specialized in community health. P2: Nurse P3: Emergency technician	Emergency medical services (Medicalized ambulance)	P1: Female/42 P2: Female/48 P3: Male/50
	TEAM 2	P4: Doctor specialized in community health. P5: Nurse P6: Emergency technician	Emergency medical services (Medicalized ambulance)	P4: Female/35 P5: Female/41 P6: Male/38
Group B (ICU)	TEAM 3	P7: Doctor specialized in intensive medicine. P8: Nurse P9: Technician specialized in nursing auxiliary care	Hospital Intensive Care Unit	P7: Female/37 P8: Female/42 P9: Female/35
	TEAM 4	P10: Doctor specialized in intensive medicine. P11: Nurse P12: Technician specialized in nursing auxiliary care	Hospital Intensive Care Unit	P10: Female/43 P11: Female/31 P12: Female/33

^1^ Teams 1 and 2 (out-of-hospital emergencies) comprised group A, and teams 2 and 3 comprised group B.

**Table 2:** Structure, composition and characteristics of the focus groups.

Group	Criteria of Suitability and Composition	Duration of the Group Interview	Researchers	Data Collection
Group (EMS)	Heterogeneity/Homogeneity: 2 doctors, 2 nurses, 2 emergency technicians	65 min	1 observer ^1^ + 1 moderator	Audiovisual with consent
Group (ICU)	Heterogeneity/Homogeneity: 3 doctors, 6 nurses, 3 Auxiliary nursing technicians	130 min	1 observer ^1^ + 1 moderator	Audiovisual with consent

^1^ The role of the observer was to help the moderator in the guidance of the group, take note of the first spoken and non-spoken impressions, and pay attention to the audiovisual equipment.

**Table 3:** Scenarios and procedures Phase 3 groups A (EMS) and B (ICU).

Team	Prebriefing	Briefing	Simulation	Debriefing	Repetition of the Simulation Scenario
Team 1 acts, (Team 2 observes)	Safe environment and familiarization with scene and material. 15 min	SBAR 5 min	Scenario with COVID patient with Coronary syndrome at home, and Cardiorespiratory arrest 15 min	35 min	15 min
REST 30 min					
Team 2 acts (Team 1 observes)	Safe environment and familiarization with scene and material. 15 min	SBAR 5 min	Scenario with Elderly woman with breathing difficulty 15 min	35 min	15 min
Team 3 acts, (Team 4 observes)	Safe environment and familiarization with scene and material. 15 min	SBAR 5 min	Scenario of Advanced Life Support with COVID patient 15 min	35 min	15 min
REST 30 min					
Team 4 acts (Team 3 observes)	Safe environment and familiarization with scene and material. 15 min	SBAR 5 min	Scenario of ICU admittance with agitated and non-collaborating patient 15 min	35 min	15 min

**Table 4:** Categories and sub-categories of the EMS focus group.

Categories	Sub-Categories	Example Verbatim Phrases
Increase in Theoretical Knowledge	Need for clear protocols at the start of the pandemic	“(…) for me, to tell you the truth, the thing that caught my attention was the improvisation, the changes from one on-call to another, for us as a unit, as well as the hospitals as the patient reception units” “(…) lots of fear on the street, fear in people with pathologies, but also a lot of misinformation. The problem is that trivial pathologies were being confused with coronavirus.”
	Information about the clinical-epidemiological characteristics and transmission of COVID-19	
	Management of disposable residues contaminated with COVID-19	“(…) we have to have set and very clear procedures about who has to check how they are removed, where they are located, where they are thrown away, and this training has to be given, especially because the more protocols there are, and the more training received, the easier it will be to avoid mistakes, everyone knows this”
	Management in the cleaning and disinfection of the ambulance	“there has been a great lack of knowledge (…) each one did what he or she knew, because as there was no protocol (…), there have been days in which the cleaning of surfaces exclusively with diluted sodium hypochlorite with immediate activation of the unit was ordered and another day the application of an aerosol in the room and the seal of the ambulance was ordered for three hours.”
	Triage/Resources in the context of the COVID-19 pandemic	“If it is an ordinary situation, triage is important, in the situation we are in, in which we had 3000 calls, triage became a very important element. The problem is, as I’ve said, that there is a great amount of triage, because right now I’m looking at the screen, and 80% of the care provided is coronavirus. The assigning of resources is very, very complicated”
Training in Technical Skills	Wearing and placement of PPEs	“the subject of putting on and taking off the PPE has not been explained either. It was explained back then with the Ebola scare, I don’t remember. I’ve put it on and taken it off was well as I could, trying to do it in the best possible manner, but self-learned”
	Removal of PPEs	
	Reanimation of COVID+ patients	“And then the management of the Covid+ patient in a CPR, with a respiratory problem, which we have not done either, just like you say, in a self-learned manner, and whatever you have read, and what you know from other experiences, and I’ve made this mistake, and I know that next time it shouldn’t happen.”
Training on Non-TechnicaL Skills and/or Attitudes	Communication-Mediation of the team with patients-family members	“(…) the training of the communication channels (…) coming together with the doctors from the coordination center, and put together what questions should be asked, in what types of cases, etc.” “(…) and about the attitudes, well, what we’ve commented about management of emotions, I think it’s basic. I have exploded and cried in many on-calls (…)” “(…) I think it would be timely to include traces of assertiveness or how to manage with perturbed individuals? In situations of conflict? Or difficult situations?” “(…) work could be done in the management of all the emotions we have seen; fear, anger, frustration, impotence, we could also work on burn out and emotional exhaustion, de-personalization, and low self-realization (…)”
	Management of emotions	
	Management of stress, to build a team (Crisis Resource Management)	
Combined Training in Technical Skills and Theoretical Knowledge	Integration of theory and practice in different situations	“(…) the placement, working with the PPE and removal of the PPE can be done using the idea of the different types of care, for example, how the severe trauma protocol changes from a normal care to care with a PPE”

**Table 5:** Categories and sub-categories of the ICU focus group.

Categories	Subcategories	Example Verbatim Phrases
Training on Theoretical Knowledge	Need for clear protocols at the start of the pandemic	“…but it was especially on the fly, improvised…trial and error. There was no planning” “…we didn’t even know how to take samples; all the information came in small drops…we flying blind”
	Information about the clinical-epidemiological characteristics and transmission of COVID-19	“(…) the first two patients who stopped with a saturation of 98%, and this is when we told ourselves…hey, this is what they say it happens” “…this is not a really bad pneumonia…it’s the other thing…”
Training in Technical Skills	Wearing and placement of PPEs	“(…) we got a talk about how to put on the PPE…but it wasn’t a PPE, it was a robe…how to put it on wasn’t very clear, we had to learn how to put it on by ourselves…”
	Removal of PPEs	“(…) No one taught me how to dress…and I don’t think YouTube is the place to train myself…”
Training on Non-Technical Skills and/or Attitudes	Communication of bad news	“…they are watching their dead family member…a young man, without even being able to touch their dad…and the answer was that….because it’s protocol…” “I remember that case and seeing the woman on the floor, … and I started to cry…but it was the mental collapse of everyone at the moment” “(…) She was in very bad shape, very distressed…she was seeing that the patient was not doing well…and she had a small child…and for me, that moment…puff… ” “(…) I remember hearing people say that they didn’t want to come to work” “It’s more about the emotional stress, the fear you have about becoming infected, and your family…especially at the start”
	Management of emotions	

**Table 6:** Results of the intervention evaluations for each of the scenarios (initial and repeated).

Scenarios	Dimension	Pre-Test	Post-Test	Bootstrap			Pre-Test–Post-Test		
		Mean (SD)-Before	Mean (SD)-After	Mean	CI 95%		*t*	*p* Value	*d* *
ICU—Agitated patient	Relief/info	3.42 (1.06)	4.33 (0.42)	0.91	−0.24	2.06	2.20	0.09	0.98
	Protection	2.63 (0.73)	3.86 (1.09)	1.22	0.09	2.37	2.99	0.04	1.33
	Management agitation	3.78 (0.63)	4.00 (0.69)	0.22	−0.17	0.61	1.58	0.19	0.70
	Use of NIMV	2.89 (0.98)	3.64 (0.76)	0.76	−0.37	1.88	1.86	0.14	0.83
	Communication	3.66 (1.01)	4.20 (0.73)	0.54	−0.38	1.47	1.63	0.18	0.73
ICU—ALS	Maneuver	3.11 (0.79)	3.95 (0.57)	0.84	−0.21	1.89	2.21	0.09	0.99
	Protection	2.71 (0.44)	3.26 (0.40)	0.54	−0.11	1.20	2.30	0.08	1.03
	Communication	2.91 (0.80)	4.03 (0.57)	1.11	−0.02	2.25	2.73	0.05	1.23
EMS—CPR	Protection	3.23 (0.38)	3.10 (0.61)	−0.13	−0.94	0.67	−0.46	0.67	0.65
	Management	3.69 (0.73)	4.36 (0.25)	0.67	−0.26	1.60	1.99	0.12	0.74
	Communication	3.11 (0.97)	4.40 (0.16)	1.29	0.14	2.43	3.12	0.04	0.92
EMS—Breathing difficulty	Protection	3.27 (0.46)	4.38 (0.26)	1.11	0.44	1.78	4.61	0.01	2.06
	Management	3.60 (0.42)	4.80 (0.20)	1.20	0.62	1.78	5.72	0.005	2.56
	Control of disruption	2.45 (0.66)	3.78 (0.26)	1.18	0.20	2.48	3.75	0.022	1.87
	Communication	2.10 (1.08)	4.40 (0.20)	2.30	0.74	3.86	4.10	0.015	1.67

* Cohen’s *d* values: 0.41 = minimum effect size representing a “practically” significant effect; 0.41–1.15 = small effect; 1.15–2.70 = medium effect; >2.70 = large effect.

## Data Availability

The data are available to those who wish them by sending an email to the corresponding authors. News about this project can be watched at https://www.youtube.com/watch?v=P4LlG2rcNJY (accessed on 23 September 2021).

## References

[1] Huang C., Wang Y., Li X., Ren L., Zhao J., Hu Y., Zhang L., Fan G., Xu J., Gu X. (2020). Clinical features of patients infected with 2019 novel coronavirus in Wuhan, China. Lancet.

[2] Zhu N., Zhang D., Wang W., Li X., Yang B., Song J., Zhao X., Huang B., Shi W., Lu R. (2020). A novel coronavirus from patients with pneumonia in China, 2019. N. Eng. J. Med..

[3] Ng K., Poon B.H., Puar T.H.K., Quah J.L.S., Loh W.J., Wong Y.-J., Tan T.Y., Raghuram J. (2020). COVID-19 and the Risk to health care workers: A case report. Ann. Intern. Med..

[4] Leal-Costa C., Díaz-Agea J.L., Ruzafa-Martínez M., Ramos-Morcillo A.J. (2021). Work-related stress amongst health professionals in a pandemic. An. Sist. Sanit. Navar..

[5] Romero C.S., Delgado C., Catalá J., Ferrer C., Errando C., Iftimi A., Benito A., de Andrés J., Otero M., The PSIMCOV Group (2020). COVID-19 psychological impact in 3109 healthcare workers in Spain: The PSIMCOV group. Psychol. Med..

[6] Pappa S., Ntella V., Giannakas T., Giannakoulis V.G., Papoutsi E., Katsaounou P. (2020). Prevalence of depression, anxiety, and insomnia among healthcare workers during the COVID-19 pandemic: A systematic review and meta-analysis. Brain Behav. Immun..

[7] Badahdah A., Khamis F., Mahyijari N.A., Balushi M.A., Hatmi H.A., Salmi I.A., Albulushi Z., Noomani J.A. (2020). The mental health of health care workers in Oman during the COVID-19 pandemic. Int. J. Soc. Psychiatry.

[8] García-Basteiro A., Alvarez-Dardet C., Arenas A., Bengoa R., Borrell C., Val M.D., Franco M., Sánchez M.G., Otero J.J.G., Valcárcel B.G.L. (2020). The need for an independent evaluation of the COVID-19 response in Spain. Lancet.

[9] Suárez-García I., López M.J.M.d.A., Vicente A.S., Abascal P.L. (2020). SARS-CoV-2 infection among healthcare workers in a hospital in Madrid, Spain. J. Hosp. Infect..

[10] d’Ettorre G., Ceccarelli G., Santinelli L., Vassalini P., Innocenti G.P., Alessandri F., Koukopoulos A.E., Russo A., Tarsitani L. (2021). Post-traumatic stress symptoms in healthcare workers dealing with the COVID-19 pandemic: A systematic review. Int. J. Environ. Res. Public Health.

[11] Clay J.M., Parker M.O. (2020). Alcohol use and misuse during the COVID-19 pandemic: A potential public health crisis?. Lancet Public Health.

[12] Xiang Y.-T., Yang Y., Li W., Zhang L., Zhang Q., Cheung T., Ng C. (2020). Timely mental health care for the 2019 novel coronavirus outbreak is urgently needed. Lancet Psychiatry.

[13] Drennan V.M., Ross F. (2019). Global nurse shortages-the facts, the impact and action for change. Br. Med. Bull..

[14] Reddy B.V., Gupta A. (2020). Importance of effective communication during COVID-19 infodemic. J. Fam. Med. Prim. Care.

[15] Lewis R., Strachan A., Smith M.M. (2012). Is high fidelity simulation the most effective method for the development of non-technical skills in nursing? A review of the current evidence. Open Nurs. J..

[16] Flin R., Maran N. (2004). Identifying and training non-technical skills for teams in acute medicine. BMJ Qual. Saf..

[17] Díaz-Agea J.L., Martín-Robles M.R., Jiménez-Rodríguez D., Morales-Moreno I., Viedma-Viedma I., Leal-Costa C. (2018). Discovering mental models and frames in learning of nursing ethics through simulations. Nurse Educ. Pract..

[18] Pires S., Monteiro S., Pereira A., Chaló D., Melo E., Rodrigues A. (2017). Non-technical skills assessment for prelicensure nursing students: An integrative review. Nurse Educ. Today.

[19] Shah A.P., Falconer R., Watson A.J.M., Walker K.G. (2021). Teaching surgical residents in the COVID-19 era: The value of a simulation strategy. J. Surg. Educ..

[20] Kolb D.A. (2014). Experiential Learning: Experience as the Source of Learning and Development.

[21] Lamé G., Dixon-Woods M. (2020). Using clinical simulation to study how to improve quality and safety in healthcare. BMJ Simul. Technol. Enhanc. Learn..

[22] Cant R.P., Cooper S.J., Lam L.L. (2020). Hospital nurses’ simulation-based education regarding patient safety: A scoping review. Clin. Simul. Nurs..

[23] Roussin C.J., Weinstock P. (2017). SimZones: An organizational innovation for simulation programs and centers. Acad. Med..

[24] Díaz-Agea J.L., Megías-Nicolás A., García-Méndez J.A., Adánez-Martínez M.d.G., Leal-Costa C. (2019). Improving simulation performance through Self-Learning Methodology in Simulated Environments (MAES©). Nurse Educ. Today.

[25] Díaz-Agea J.L., Jiménez-Rodríguez D., García-Méndez J.A., Hernández-Sánchez E., Sáez-Jiménez A., Leal-Costa C. (2017). Patient-Oriented debriefing: Impact of real patients’ participation during debriefing. Clin. Simul. Nurs..

[26] Juguera-Rodríguez L., Pardo-Ríos M., Castillo-Hermoso M., Pérez-Alonso N., Leal-Costa C., Díaz-Agea J.L. (2020). Impact of simulation-based learning on family caregivers during the rehabilitation period of individuals with spinal cord injury. Spinal Cord.

[27] Borgmeyer A., Gyr P.M., Ahmad E., Ercole P.M., Balakas K. (2017). Pediatric nurse practitioners effective in teaching providers the Asthma Action Plan using simulation. J. Pediatr. Nurs..

[28] Karageorge N., Muckler V.C., Toper M., Hueckel R. (2020). Using simulation with deliberate practice to improve pediatric ICU nurses’ knowledge, clinical teamwork, and confidence. J. Pediatr. Nurs..

[29] Merandy K., Chambers L.L., Morgan K., Cane D., Barimah L.M., Vasquez M., Rosenberg J. (2021). Simulation based educational intervention for enhancing irrigation skills of nurses caring for patients with cutaneous continent urinary diversions and orthotopic neobladders. J. Wound Ostomy Cont. Nurs..

[30] Bohmann F.O., Gruber K., Kurka N., Willems L.M., Herrmann E., de Rochemont R.d.M., Scholz P., Rai H., Zickler P., Ertl M. (2021). Simulation-based training improves process times in acute stroke care (STREAM). Eur. J. Neurol..

[31] Lim W.Y., Ong J., Vimal V., Lim H., Tan H.C., Wong P., Ho V.K., Ong S.G.K. (2021). High-fidelity simulation training with PPE may optimise resuscitation outcomes in the COVID-19 era. Resuscitation.

[32] Foong T.W., Ng E.S.H., Khoo C.Y.W., Ashokka B., Khoo D., Agrawal R. (2020). Rapid training of healthcare staff for protected cardiopulmonary resuscitation in the COVID-19 pandemic. Br. J. Anaesth..

[33] Andreae M.H., Dudak A., Cherian V., Dhar P., Dalal P.G., Po W., Pilipovic M., Shah B., Hazard W., Dl R. (2020). Healthcare simulation to prepare for the COVID-19 pandemic. J. Clin. Anesth..

[34] Li D.-F., Shi C.-X., Shi F.-Z., Zhao L., Zhao R., Kang W.-Q. (2020). Effects of simulation training on COVID-19 control ability and psychological states of nurses in a children’s hospital. Eur. Rev. Med. Pharmacol. Sci..

[35] (2021). Practical Advice from the Frontline of SARS-CoV-2 Outbreak. European Society of Paediatric Neonatal Intensive Care—ESPNIC. https://espnic-online.org/News/Latest-News/Practical-advice-from-the-frontline-of-SARS-CoV-2-outbreak.

[36] Pan D., Rajwani K. (2021). Implementation of simulation training during the COVID-19 pandemic: A New York hospital experience. Simul. Healthc..

[37] Lioce L., Meakim C.H., Fey M.K., Chmil J.V., Mariani B., Alinier G. (2015). Standards of best practice: Simulation standard IX: Simulation design. Clin. Simul. Nurs..

[38] (2016). INACSL Standards of Best Practice: Simulation^SM^ Simulation Design. Clin. Simul. Nurs..

[39] Alinier G. (2011). Developing high-fidelity health care simulation scenarios: A guide for educators and professionals. Simul. Gaming.

[40] D’Zurilla T.J., Goldfried M.R. (1971). Problem solving and behavior modification. J. Abnorm. Psychol..

[41] Phrampus P.E., O’Donnell J.M., Levine A.I., DeMaria S., Schwartz A.D., Sim A.J. (2013). Debriefing using a structured and supported approach. The Comprehensive Textbook of Healthcare Simulation.

[42] Bland J.M., Altman D.G. (2015). Statistics notes: Bootstrap resampling methods. BMJ.

[43] Ferguson C.J. (2016). An effect size primer: A guide for clinicians and researchers. Methodological Issues and Strategies in Clinical Research.

[44] Fleiss J.L., Levin B., Paik M.C. (2013). Statistical Methods for Rates and Proportions.

[45] Streiner D.L., Norman G.R., Cairney J. (2015). Health Measurement Scales: A Practical Guide to Their Development and Use.

[46] Nayahangan L.J., Konge L., Russell L., Andersen S. (2021). Training and education of healthcare workers during viral epidemics: A systematic review. BMJ Open.

[47] Rajakaruna S.J., Liu W.-B., Ding Y.-B., Cao G.-W. (2017). Strategy and technology to prevent hospital-acquired infections: Lessons from SARS, Ebola, and MERS in Asia and West Africa. Mil. Med. Res..

[48] Carvalho E., Castro P., León E., Del Río A., Crespo F., Trigo L., Fernández S., Trilla A., Varela P., Nicolás J.M. (2019). Multi-professional simulation and risk perception of health care workers caring for Ebola-infected patients. Nurs. Crit. Care.

[49] Abrahamson S.D., Canzian S., Brunet F. (2006). Using simulation for training and to change protocol during the outbreak of severe acute respiratory syndrome. Crit. Care.

[50] Hernández E., Camacho M., Leal-Costa C., Ruzafa-Martínez M., Ramos-Morcillo A.J., Cazorla E., Díaz-Agea J.L. (2021). Does multidisciplinary team simulation-based training improve obstetric emergencies skills?. Healthcare.

[51] Yule S., Smink D.S. (2020). Nontechnical skill countermeasures for pandemic response. Ann. Surg..

[52] Schaumberg A., Schröder T., Sander M. (2017). Emergency medical training through simulation: Always the same for everyone?. Anaesthesist.

[53] Foster M., Gilbert M., Hanson D., Whitcomb K., Graham C. (2019). Use of simulation to develop teamwork skills in prelicensure nursing students: An integrative review. Nurse Educ..

[54] Alsabri M., Boudi Z., Lauque D., Roger D.D., Whelan J.S., Östlundh L., Allinier G., Onyeji C., Michel P., Liu S.W. (2020). Impact of teamwork and communication training interventions on safety culture and patient safety in emergency departments: A systematic review. J. Patient Saf..

[55] Carne B., Kennedy M., Gray T. (2012). Review article: Crisis resource management in emergency medicine. Emerg. Med. Australas..

[56] Fanning R.M., Goldhaber-Fiebert S.N., Udani A.D., Gaba D.M., Levine A.I., DeMaria S., Schwartz A.D., Sim A.J. (2013). Crisis resource management. The Comprehensive Textbook of Healthcare Simulation.

[57] Guimond M.E., Sole M.L., Salas E. (2009). TeamSTEPPS. Am. J. Nurs..

[58] Clancy C.M., Tornberg D.N. (2007). TeamSTEPPS: Assuring Optimal Teamwork in Clinical Settings. Am. J. Med. Qual..

